# Mobile health applications in self-management of patients with chronic obstructive pulmonary disease: a systematic review and meta-analysis of their efficacy

**DOI:** 10.1186/s12890-018-0671-z

**Published:** 2018-09-04

**Authors:** Fen Yang, Yuncui Wang, Chongming Yang, Hui Hu, Zhenfang Xiong

**Affiliations:** 1 School of Nursing, Hubei University of Chinese Medicine, Wuhan, China; 2 Research Support Center, Brigham Young University, Provo, UT USA

**Keywords:** Chronic obstructive pulmonary disease, Self-management, Hospital admissions, Mobile applications

## Abstract

**Background:**

Mobile health applications are increasingly used in patients with Chronic Obstructive Pulmonary Disease (COPD) to improve their self-management, nonetheless, without firm evidence of their efficacy. This meta-analysis was aimed to assess the efficacy of mobile health applications in supporting self-management as an intervention to reduce hospital admission rates and average days of hospitalization, etc.

**Methods:**

PubMed, Web of Science (SCI), Cochrane Library, and Embase were searched for relevant articles published before November 14th, 2017. A total of 6 reports with randomized controlled trials (RCTs) were finally included in this meta-analysis.

**Results:**

Patients using mobile phone applications may have a lower risk for hospital admissions than those in the usual care group (risk ratio (RR) = 0.73, 95% CI [0.52, 1.04]). However, there was no significant difference in reducing the average days of hospitalization.

**Conclusion:**

Self-management with mobile phone applications could reduce hospital admissions of patients with COPD.

## Background

Chronic Obstructive Pulmonary Disease (COPD) is a major global chronic disease which affected millions of people worldwide [1], causing considerable hospital admissions. The World Health Organization (WHO) has estimated that COPD which causes considerable hospital admission will become the third cause of global deaths by 2020 [2–5]. These patients are heavy users of healthcare and social service resources [6, 7]. As there is currently no cure for COPD, appropriate self-care and management may play an important role in the patients’ lifetime. Self-management techniques, such as adherence to medication, exercises, and prompt medical care, are crucial to improve the health status and have the potential to reduce hospital admissions [8–11].

Mobile health (mHealth), is now widely used for self-management of COPD, a term used to describe medical practice and healthcare in support of mobile computing and mobile devices (such as tablets, mobile phones, etc.). However, it is unclear whether these applications are beneficial to patients [12]. The deployment of eHealth applications is conducive to the availability of health care, which in turn enhances the patient’s understanding of his illness, sense of control, and willingness to manage himself [13]. However, cheaper and widely available mobile phones are not other specialized medical devices. Mobile phones with applications to monitor, prompt, and record health behaviors have become a feasible and acceptable intervention [14]. Some reviews reported that mHealth applications were effective in promoting disease self-management and daily lifestyle changes [15–17]. Several studies showed that mobile phones could deliver effective behavior change interventions and had many positive evidences [9, 18–20]. Mobile phones were also found effective in promoting COPD patients’ physical activity and exercise capacity [21]. However, another study found that COPD patients with telephone-based care had greater mortality than usual care [22]. It is not clear how effectively mobile phone interventions could improve hospital admissions and lengths of hospitalization of COPD patients. Therefore, this study was aimed to compare the efficacy of mobile phone intervention with usual care in self-management, in terms of hospital admissions and the lengths of hospitalization.

## Methods

### Data sources and searches

A literature search without language restriction was performed using PubMed, Web of Science, the Cochrane Library, and Embase databases to identify potentially eligible studies published prior to November 14, 2017. All titles, keywords, and abstracts were examined in accordance with our search criteria. Full reports also were reviewed in case of uncertainty. In addition, references of retrieved studies and review articles were also manually checked to identify additional relevant studies. Some authors were even contacted for further information.

### Study selection

Each study had to meet four criteria to be included in this study. First, studies were RCTs reported in full text with a title and abstract. Second, it included adults with a clinical diagnosis of COPD and compare mobile phone application interventions with the control group in usual care only (namely, routine or standard care). Third, telemonitoring studies entailed a self-management by COPD patients with ≥1 month follow-up. Fourth, the trials evaluated at least one of the following primary or secondary outcomes. The primary outcome was a hospital admission. The secondary outcome was the length of hospitalization, activity level, and lung function (e.g., predicted FEV1 percentage). Inclusion of each study was evaluated and determined independently by two reviewers. Exclusion criteria included: (1) reports based on systematic reviews and meta-analyses; (2) mobile-based interventions only via phone calls or sending messages.

### Data extraction and quality assessment

From the articles that met the inclusion criteria, two reviewers independently extracted descriptions of the objectives, design, participants, interventions, and follow-up time. Any disagreements in data extraction were resolved by a discussion among the reviewers, and a final decision was made by another reviewer. If it is difficult or unclear to extract data from an article, its author was directly contacted to request the original data.The Cochrane Group’s predesigned table [23] was used to assess the quality of the studies, including randomization, allocation concealment, similarity of baseline, criteria of inclusion/exclusion, blinding of participants and researchers, blinding of assessors, attrition rates, reporting of lost participants, and other sources of biases. Studies were scored one point for each fulfilled criterion. The quality of the studies was divided into three levels: low (≤3 points), moderate (4–6 points), and high (7–9 points).

### Data synthesis and analysis

Eight studies were selected for the systematic review [24–31] and six of that were included for the meta-analysis with a random-effects model [24–29]. The outcomes reported by similar multiple studies were combined for the analysis. Also, meta-analyzed were the RCTs that reported the number of readmissions and the average days of hospitalization of each group (usual care vs. eHealth).

Data were obtained from the original selected studies or calculated [32] from the raw data. Risk ratios (RRs) were calculated for hospital admissions and mortality rates. Statistical heterogeneity was measured with the chi-square (*χ*^2^) and *I*^2^ statistics whose values greater than 50% indicate a high heterogeneity for the latter [33]. Publication bias was depicted with Begg’s plot. Standard Mean Differences (SMD) were estimated with random effects modeling. All analyses were performed using Stata 12.0.

## Results

### Basic characteristics of the studies

**Fig. 1:**
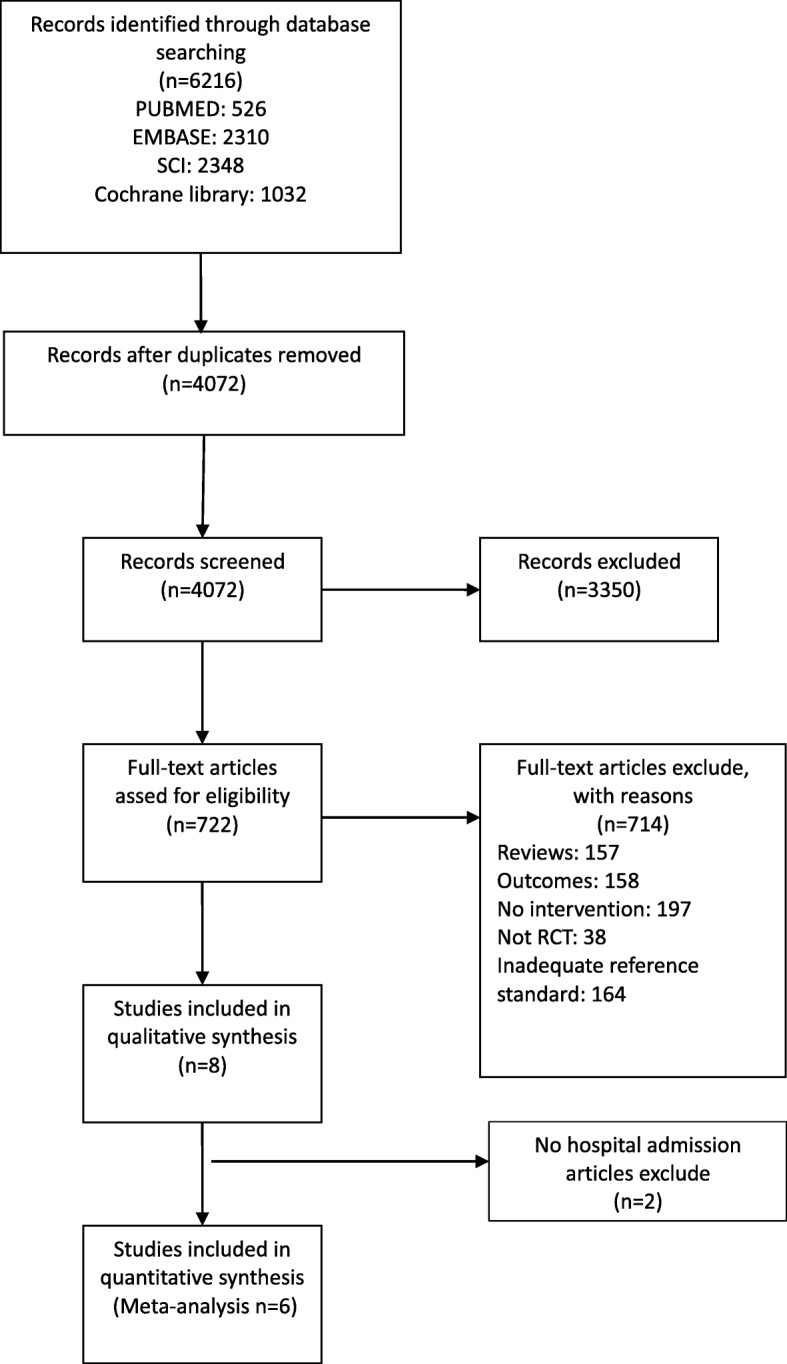
Summary of Evidence Search and Publication Selection

From the 4072 potentially relevant reports initially identified, 3350 publications were excluded. The remainder of 722 retrieved reports were selected for full-text assessments and detailed evaluations. Finally, eight articles fulfilled our inclusion criteria [24–31] and only six articles were included in our meta-analysis [24–29] because two didn’t report the patients’ hospital admission [30, 31]. Figure 1 shows the literature flow diagram. We also extracted some additional information, such as country, mean age, the sample size of each group, sex, FEV1, the intervention methods, the length of follow-up and BMI, as shown in Table 1. Most RCTs compared a continued care with a usual care. Six RCTs reported the primary outcome of hospital admissions [24–29]. Only one study was a multicenter RCTs, and the others were conducted in single centers. There were totally 391 participants with COPD, 293 (74.9%) of whom were men. The participants in one study had additional heart failures [27]. The sample sizes of subjects ranged from 24 to 99. There were six studies reporting the duration of the intervention equal or more than 6 months. The age of participants ranged from 63.5 to 81.0 years. The lung dysfunction was indicated by FEV1 (% predicted) that ranged from 37.9 to 58.9%. BMI ranged from 23.2 to 28.8 kg/m^2^. Follow-ups were conducted for a range of 1 to 12 months and a mean of 6.9 months.

**Table 1:** Description of included trails

Source	Country	Mean (Age, y)	N	Setting	Male (%)	FEV1%	BMI (kg/m^2^)	Months of follow-up	Intervention
Liu WT et al., 2008 [26]	China	72.1	IG:24;CG:24	Single-center	48(100.0)	45.6	23.2	12	Patients were asked to complete respiratory symptoms by a cell phone using a Java application software, before they started daily endurance walking training.
Chau JP et al., 2012 [25]	China	72.9	IG:22;CG:18	single-center	39(97.5)	37.9	NR	2	The ASTRI telecare system (AST): a device kit which includes a mobile phone, a respiratory rate sensor and a pulse oximeter, an online network platform, a call center and a networking system. Participants in the intervention group measured their oxygen saturation, pulse rate and respiration rate at home and sent the results to the online network platform by mobile phone.
Jehn M et al., 2013 [24]	Germany	66.6	IG:32;CG:30	single-center	48(74.4)	51.4	27.3	9	Tele-monitoring: COPD Assessment Test (CAT), daily lung function and weekly 6-minutte walk test (6MWT). The patients entered CAT by mobile phone (PDA system, MMA400).
Martín-Lesende I et al., 2013 [27]	Spain	81.0	IG:28;CG:30	Multicenter	34(58.6)	NA	NR	3, 6, 12	Tele-monitoring: daily transmissions from the patients’ homes of the following self-measured clinical parameters (such as blood oxygen saturation, blood pressure, heart and respiratory rates, body weight and temperature) using a smart phone- personal digital assistant (PDA).
Pedone C et al., 2013 [28]	Italy	74.8	IG:50;CG:49	Single-center	67(67.7)	54.0	NR	9	“SweetAge” monitoring system: A commercial cellular telephone was equipped with a software that allowed the reception of the data(oxygen saturation, heart rate, near-body temperature, overall physical activity) transmitted by the wristband and sent the data to the monitoring system.
Tabak M et al., 2014 [29, 30]	the Netherlands	63.5	IG:12;CG:12	Single-center	12(50.0)	43.0	26.8	9	Condition Coach: teleconsultation (module for comments and asking questions of the patient’s primary care physiotherapist and vice versa), Web-based exercising (including breathing exercises, relaxation, mobilization, resistance and endurance training, and mucus clearance), self-management and activity coach(A smartphone shows the measured activity cumulatively in a graph).
Tabak M et al., 2014 [29, 30]	the Netherlands	66.6	IG:14;CG:16	Single-center	19(63.3)	52.6	28.8	1	Tele-rehabilitation: (1) a smartphone (HTC P3600/3700) was used for activity coach; (2) web portal with a symptom diary for self-treatment of exacerbations and an overview of the measured activity levels.
Wang CH et al., 2014 [31]	China	71.7	IG:14;CG:16	Single-center	26(86.7)	58.9	23.5	6	Patients in the intervention group performed daily endurance exercise training under mobile phone guidance, and adherence was reported back to the central server.

*NR* not reported, *IG* Intervention Group, *CG* Control Group

The intervention of the five studies included mobile/smartphones with different software (Phone-personal digital assistant, MMA400, HTC P3600/3700, Sony Ericsson K600i, and HTC Desire S) and measuring devices. Applications were installed on smartphones to support patients in recording and monitoring their own physiological status, such as oxygen saturation levels, pulse rates, pedometers, or blood pressure monitors; or health behaviors, such as medications and dietary intake and exercise levels. Patients uploaded the data to their phones and sent the data to the networking/monitoring systems for healthcare providers to follow up and personalize feedbacks. The aim of the intervention was to train patients and promote self-monitoring and healthy lifestyle behaviors. In three of all the trials, the mobile/smartphone was used to coach activities. The patients in control groups received usual care.

### Study quality and publication Bias

The overall quality of the studies (Table 2) was moderate to high (4–7 scores). Five studies scored less than or equal to 6, and three studies scored less than 9. The most common reason for lower scores was the absence of a double–blind procedure, which was impossible due to the nature of the intervention. The assessors were not blinded to the outcomes in all the studies (100.0%) and researchers/participants were not blinded in 6 studies (75.0%). Only one study (12.5%) did not report the characteristics of participants lost for follow-ups. Begg’s plot that was used to examine publication bias showed that there was no evident publication bias (*p* = 1.00).

**Table 2:** Quality assessment of included studies

Study	Randomization	Concealing Allocation	Baseline Similarity	Inclusion/Exclusion Criteria	Blinded Researchers/Participants	Blinded Assessors	Attrition Rate Reported	Describing Lost Participants	Intention-to-Treat Analysis	Power Analysis	Total
Liu WT et al., 2008 [26]	Yes	No	Yes	Yes	No	No	Yes	Yes	No	Yes	6
Chau JP et al., 2012 [25]	Yes	No	Yes	Yes	No	No	Yes	Yes	No	No	5
Jehn M et al., 2013 [24]	Yes	No	Yes	Yes	No	No	Yes	No	No	No	4
Martín-Lesende I et al., 2013 [27]	Yes	No	Yes	Yes	No	No	Yes	Yes	No	Yes	6
Pedone C et al., 2013 [28]	Yes	No	Yes	Yes	No	No	Yes	Yes	Yes	Yes	7
Tabak M et al., 2014 [29, 30]	Yes	Yes	Yes	Yes	Yes	No	Yes	Yes	No	No	7
Tabak M et al., 2014 [29, 30]	Yes	Yes	Yes	Yes	Yes	No	Yes	Yes	No	No	7
Wang CH et al., 2014 [31]	Yes	No	Yes	Yes	No	No	No	Yes	No	No	4

### Hospital admission rates

**Fig. 2:**
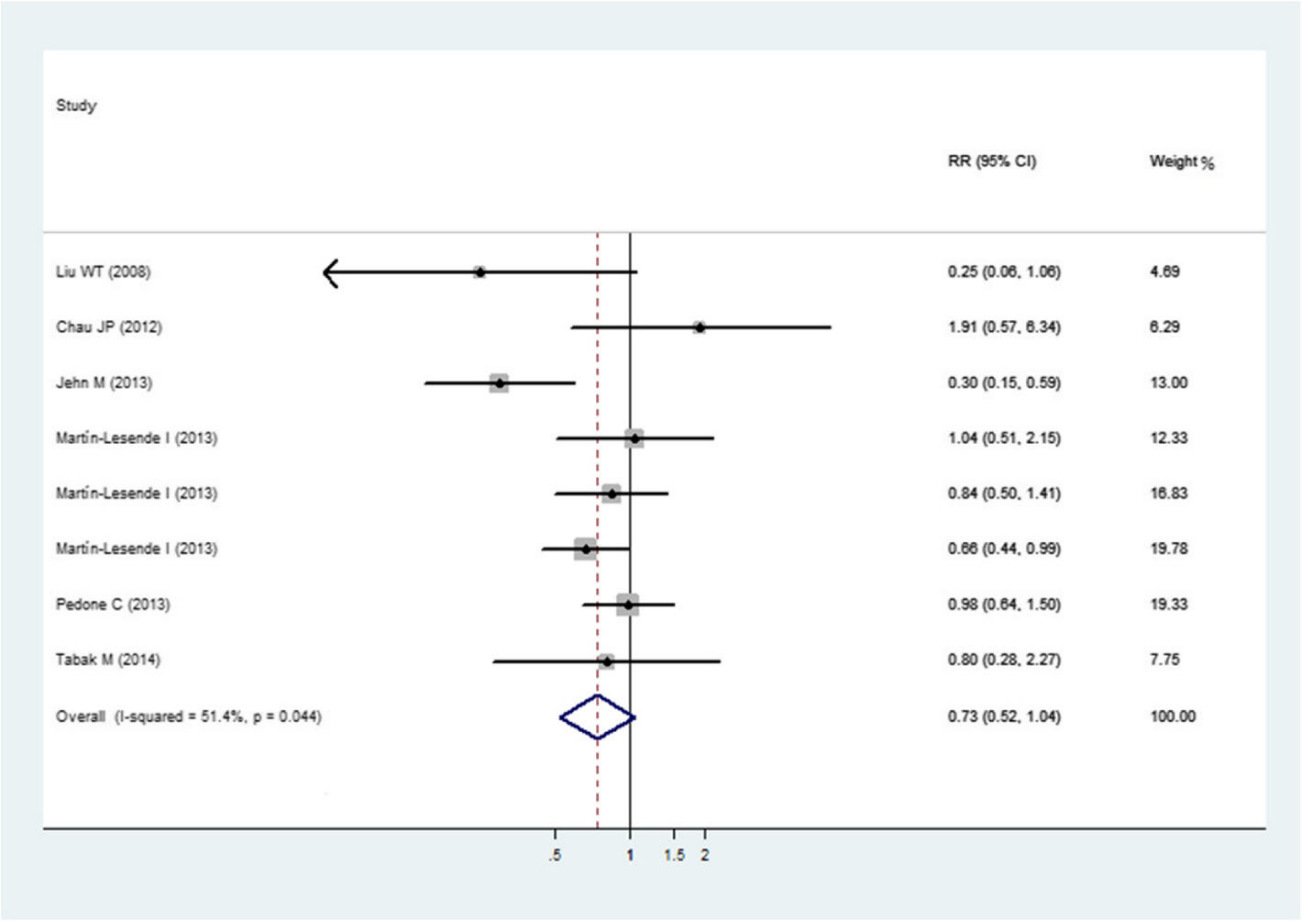
Hospital admission for Intervention Group Compared with Control Group. Weights were from the random effects analysis

Figure 2 presented our meta-analyses and RR calculations of RCTs reported hospital admission. Six studies [24–29] assessed the effect of mobile health applications on hospital admission. As Martín-Lesende [27] reported the data of patients at the follow-up of 3 months, 6 months, and 12 months, we treated it as three separate experiments in our meta-analysis. We found that a lower risk for hospital admission among patients using mobile phone applications than that of the usual care group (RR = 0.73 [95% CI, 0.52 to 1.04]). The study by Jehn M [24] found that the hospital admission rates decreased significantly (RR, 0.30 [95% CI, 0.15 to 0.59]). But the heterogeneity in the overall pooled effect is 51.4% (I^2^ = 51.4%, *p* = 0.04), implying that effect sizes varied across studies.

### Average days of hospitalization

**Fig. 3:**
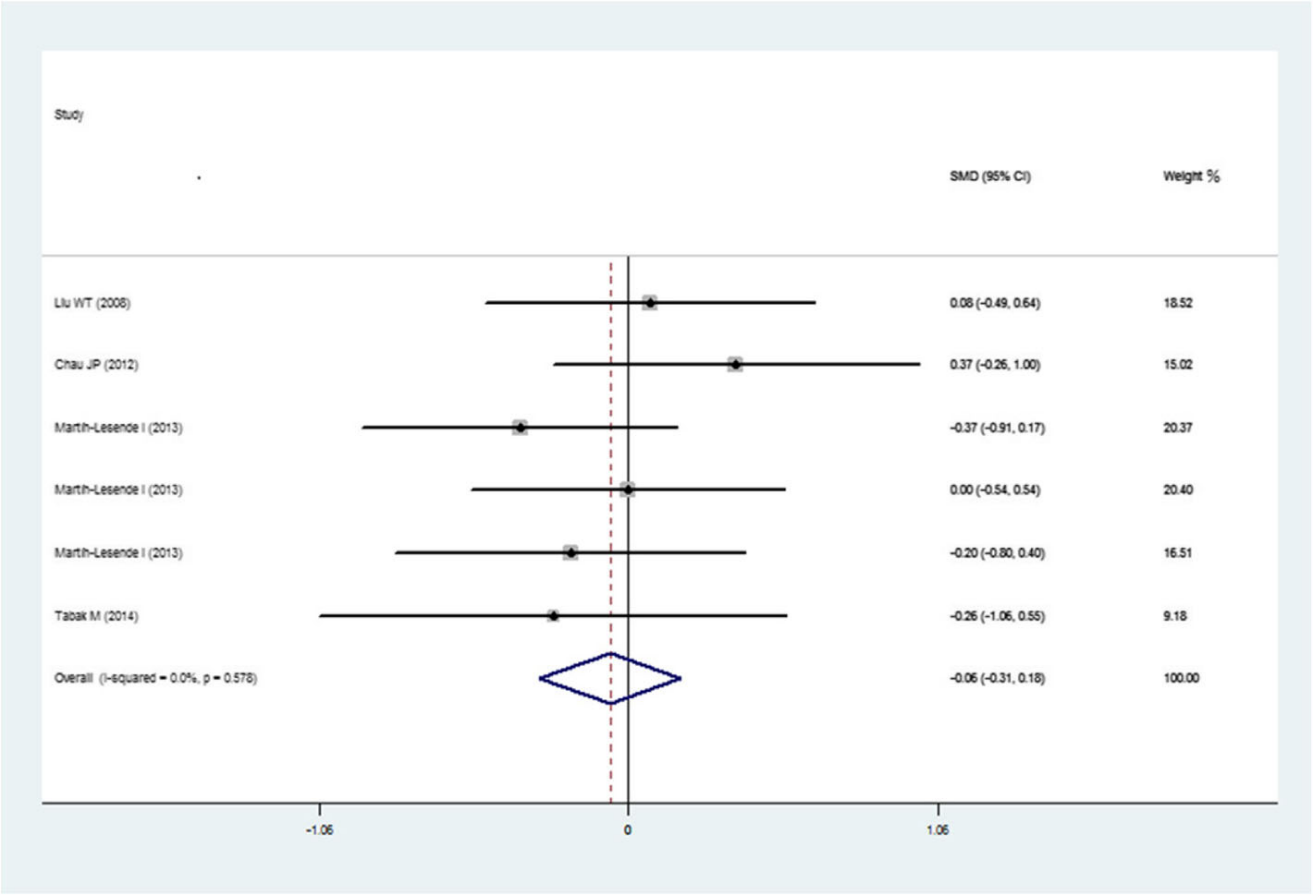
Average hospital staying days for Intervention Group Compared with Control Group. Weights are from the random effects analysis

As shown in Fig. 3, six studies reported the average days of hospital stays. No significant difference was found between the intervention group and control group (SMD -0.06 [95% CI, − 0.31 to 0.18]).

### Other results

Five articles reported that phone-based system could significantly improve exercise capacity and activity levels [24, 26, 29–31]. One study showed a significant reduction (lower predicted FEV1 percentage) in lung function of tele-monitoring intervention groups [24]. However, there was no significant differences found in another study [26].

### Sensitivity analysis

A sensitivity analysis was performed for the primary outcome to test an overall pooled effect. The results were no different between fixed and random statistical effects (RR = 0.73; *P* = 0.000). The effect of sequentially omitting a low-quality study [24] and recalculating the pooled estimates for the remaining studies did not significantly alter the effect on all cause readmission (RR = 0.73 vs. 0.83; *P* = 0.000).

## Discussion

Our results showed that mobile phone-based health applications in self-management currently could reduce hospital admissions of patients with COPD and could improve exercise capacity and activity levels, but could not reduce the average days of hospitalization.

Our findings were slightly different from another telemonitoring study which used a touch screen telemonitoring equipment to record and transmit a daily questionnaire about symptoms and corresponding treatment, and did not provide any convincing evidence of effectiveness on hospital admission and the duration of admissions [34]. The inconsistence may be due to the difference between screen telemonitoring and mobile phone. Mobile phone-based applications were found easy to learn and use by the participants as well as the patients with COPD. Mobile phone-based health applications could be practically more feasible as an intervention. Some studies reported they were a simple, reliable, easy to perform, and cost-saving intervention in behavior-changes, with advantages like adherence and intensity of the interventions, and more willingness of patients to use than other electronic devices [35, 36]. In addition, the virtual link created by sending self-monitoring data to a research nurse provided patients with a sense of continuity of care [37].

Mobile-phone-based system provides a feasible, efficient exercise training in improving exercise capacity, which was similar with other studies [38, 39]. Patients with COPD have decreased exercise capacity in their daily activities and they may have an inactive lifestyle [40–43]. Mobile phone applications, as a feasible and acceptable method for patients with COPD, can increase the capacity of self-management and the exercise adherence [44]. This result was similar with another review which evaluated the effectiveness of interventions delivered by computer and by mobile technology versus face-to-face or hard copy/digital documentary-delivered interventions [45]. A recent systematic review reported that mobile-based exercise programs could improve exercise capacity in patients with COPD in short and long term [30].

There are several limitations of our study. First, most RCTs compared an intervention with “usual care” whose details were not reported. Second, we only selected studies that reported the proportion of hospital admitted patients, but ignored information about secondary outcomes such as costs. Third, only eight studies were included in this systematic review and 6 studies in meta-analysis, most interventions were performed in short terms which could have influenced the results. Mobile phones will continue to evolve and are expected to be robust ubiquitous devices in the future, and researchers may think about how mobile phones can be used in future self-management of chronic disease. With the development of information technology and the expansion of mobile applications in medicine, we could improve the designs and clarify the effects of mobile health applications interventions for reducing hospital admission in patients with COPD in the future.

## Conclusions

The effectiveness of self-management with mobile or smart phones may help to reduce hospital admissions or improve health status of patients with COPD. Mobile phone with convenient applications have a great potential to minimize health problems and improve healthcare delivery.

## Abbreviations

COPD: Chronic obstructive pulmonary disease
mHealth: Mobile health
RCTs: Randomized controlled trials
RRs: Risk ratios
SCI: Web of science
